# Autoimmunity, dendritic cells and relevance for Parkinson’s disease

**DOI:** 10.1007/s00702-012-0842-7

**Published:** 2012-06-15

**Authors:** E. Koutsilieri, M. B. Lutz, C. Scheller

**Affiliations:** Institute of Virology and Immunobiology, University of Würzburg, Versbacher Straße 7, 97078 Würzburg, Germany

**Keywords:** Parkinson, Dendritic cells, Autoimmunity, Immune, Neuromelanin

## Abstract

Innate and adaptive immune responses in neurodegenerative diseases have become recently a focus of research and discussions. Parkinson’s disease (PD) is a neurodegenerative disorder without known etiopathogenesis. The past decade has generated evidence for an involvement of the immune system in PD pathogenesis. Both inflammatory and autoimmune mechanisms have been recognized and studies have emphasized the role of activated microglia and T-cell infiltration. In this short review, we focus on dendritic cells, on their role in initiation of autoimmune responses, we discuss aspects of neuroinflammation and autoimmunity in PD, and we report new evidence for the involvement of neuromelanin in these processes.

## Theories on initiation of autoimmunity

Autoimmunity refers to the immunological destruction of the own cells and tissues due to the failure of the organism to recognize these as self. Autoimmune diseases cover a great variety of symptoms reaching from diffuse inflammatory symptoms involving components of the innate immune system to highly antigen-specific T- and B-lymphocyte responses. Both types of autoimmunity can appear restricted to defined structures in local tissues or systemically (McGonagle and McDermott 2006). Genetic predisposition to overshooting immunity either by a loss of tolerance to self-antigens (e.g. by deficit of thymic selection, regulatory T cells) or increased sensitivity thresholds (e.g. by increased immune receptor signaling) may explain why some but not all individuals develop autoimmunity at one point of their life time. However, it cannot explain why the symptoms start at a certain time point. Therefore, additional environmental factors such as noxes, injury or infections have been discussed to trigger the loss of self-tolerance and thereby the onset of disease (Bach 2005; Chervonsky 2010). The same recognition system responsible to initiate anti-microbial immune responses against foreign antigens may then be triggered in a bystander fashion against harmless auto-antigens or the infectious environment may modify auto-antigens into chemically altered self-antigens that then appear as foreign antigens.

Evolutionarily conserved pathogen-associated molecular patterns (PAMPs) on microbes or danger-associated molecular patterns (DAMPs) released after tissue damage are recognized by different families of immune receptors, summarized under the name pattern recognition receptors (PRRs), with the toll-like receptors (TLRs) as their most prominent representatives (Mills 2011). Self-antigens may also bind PRRs under certain circumstances. First, genetic alterations in such receptors or other immune-related genes could lower the threshold for immune activation against harmless self-antigens. Second, cross-reactivity of microbial structures with self-antigens (molecular mimicry) may occur (Chastain and Miller 2012) and, finally, exogenous noxes or infections by viruses (Bianchi et al. 2007; Ferri et al. 2008; Lunemann and Munz 2007; Munz et al. 2009), bacteria (Root-Bernstein et al. 2009) or fungi (Romani 2008) may be responsible for chronic inflammatory processes promoting auto-aggression via bystander activation or epitope-spreading (Delogu et al. 2011; Kamradt and Mitchison 2001).

### Glycolipids as a source of autoantigens

More recent data indicate that proteins may in fact represent only a minor source of antigens that contribute to molecular mimicry. With the increasing identification of C-type lectin receptors as PRRs for sugars and lipids, their role as auto-antigens turned into the center of attention (Buzas et al. 2006). Probably the best example for molecular mimicry comes from glycolipid recognition in the Guillian–Barré syndrome (GBS). Antibodies against *Campylobacter jejuni* gangliosides cross react with some human gangliosides, mostly GM1 and GD1 (Hughes and Cornblath 2005; Nores et al. 2008).

The antibodies found are IgG type produced only after the isotype switch of B cells (Yuki and Odaka 2005), which strictly require CD4^+^ T-cell help. T-cell activation of dendritic cell (DC) can occur by *C. jejuni* gangliosides, but presentation of the glycolipids on MHC class II molecules is not possible (Kuijf et al. 2010). How can IgG antibodies then be generated against glycolipids that are not presented on MHC II molecules to differentiate T helper cells? NKT cells recognize glycolipids and can produce similar cytokine patterns as CD4^+^ T cells that are involved in B-cell cytokine switches (Brigl and Brenner 2004). They may be substitute the classical CD4^+^ T-helper cells as shown after injection of mice with α-galactosylceramide, a prototype glycolipid antigen for NKT cells (Lang et al. 2006) and thereby help to generate glycolipid-specific IgG antibodies without antigen-specific CD4^+^ T-cell help. Alternatively, soluble factors present in the supernatant of the glycolipid-activated DCs may directly be able to circumvent both T cell and NKT cell help (Kuijf et al. 2010).

### Oxidized glycolipids as altered self-antigens

Despite the fact that the CNS is the target organ for auto-reactive T cells in multiple sclerosis (MS), the T-cell priming event is postulated to occur in peripheral tissues (Goverman 2009). Whether these primed T cells and subsequently B cells have been primed directly against CNS antigens is unclear, although there is some evidence (Obermeier et al. 2011). It is also conceivable that they responded to a virus infection, where specific viruses gained access to the CNS. Plasma cells may then enter the CNS. Especially for EBV, higher IgG antibody titers had been measured in cerebrospinal fluid as compared to peripheral blood (Haahr and Hollsberg 2006), potentially indicating that a cerebral infection would be target also for a T-cell response. The intrathecal demonstration of oligoclonal IgG bands from MS patients by electrophoretic profiling can be used for diagnosis. However, the simultaneous increase of IgGs against different viruses may rather indicate a generalized inflammatory reponse, because infections enhance only monospecific IgGs directed against the pathogen (Boucquey et al. 1990; Sindic et al. 1990). In fact, binding of these antibodies to viral target structures in the CNS has not been demonstrated. Nevertheless, indirect microbial promotion of autoimmunity is highly evident, as, for example, impressively shown by clear influence of intestinal tract commensals on experimental autoimmune encephalomyelitis (EAE), a murine model for the early inflammatory stages of MS (Berer et al. 2011). Together, a definitive proof, which directly links virus infections with CNS autoimmunity, is still lacking.

More recent data indicate that cerebrospinal fluid of MS patients also contains increased levels of selected glycolipids such as sulfatide and, interestingly, oxidized cholesterol and phosphocholine as well as asialo GM1 when compared to healthy controls (Kanter et al. 2006). Sulfatide has been shown to associate with CD1d antigen-presenting molecules of mice (Zajonc et al. 2005) and to enhance the severity of EAE (Kanter et al. 2006). Thus, also in MS rather glycolipids than proteins might represent targets of autoimmune attack, especially when oxidation of glycolipids converts them to altered self-antigens.

## Relevance of dendritic cells (DCs) in autoimmunity

DCs are heterogenous antigen-presenting cells of the immune system that play an important role in the initiation of innate and adaptive immune responses. From one side, DCs are being considered as inflamers of immune response against microbial pathogens but also unwanted organ graft rejection and autoimmunity, on the other side they are supposed to induce and even maintain tolerance to antigens (Morelli and Thomson 2007; Steinman and Nussenzweig 2002). Tolerogenic or immunogenic functions of DCs depend on their stage of differentiation/maturation but are independent of hematopoietic origin or subset classification (Thomson and Robbins 2008). Some authors claim that the endogenous environment itself may generate factors, which decide for an immune response initiated by the DCs or the maintenance of tolerance (Matzinger 2002). Although immature mDCs capture and process antigens to present them to naïve T cells to low extends, effector T cells are not generated by them and rather tolerogenic mechanisms such as T-cell anergy or induction of regulatory T cells dominate to downregulate immune responses. These DCs can inhibit alloantigen-specific T-cell responses, reverse autoimmune diseases in murine models and induce antigen-specific T-cell tolerance (Thomson and Robbins 2008). In contrast, following a powerful immunological stimulus (such as contact with transplants or allergens, products associated with microbes or inflammation) immature DCs become mature and migrate to the respective lymph node, prime and stimulate expansion of antigen-specific T cells, and present intact proteins to B cells for their activation and subsequent antibody production (Cravens and Lipsky 2002). Activated T cells and antibodies are carried by blood to affected tissues. In autoimmune responses, these attack host proteins.

Dendritic cells also regulate immune responses against self-antigens via mechanisms such as differentiation of T-regulatory cells, T-cell anergy and clonal deletion of effector T cells which are specific for such antigens (Platt and Randolph 2010). Autoimmunity happens in environments where these regulatory mechanisms fail to control T-cell responses directed against the self-antigens. Whereas subclinical forms of autoimmunity are frequent processes, prolonged activation of autoreactive lymphocytes is requested for the development of an autoimmune disease and accompanies ongoing tissue damage (Ludewig et al. 2001). Although genetic components predispose people or animals for autoimmune diseases, trauma or tissue injury further contributes to promote autoimmunity through DAMPs (Manfredi et al. 2009; van Duivenvoorde et al. 2006). The onset of autoimmune diseases, however, is associated with viral and bacterial infections (Regner and Lambert 2001), which either trigger (Miller et al. 1997) or accord to relapses in autoimmune diseases (Andersen et al. 1993). Manifested autoimmunity may also depend on the number of DCs presenting self-antigens and the duration of antigen presentation by DCs, suggesting a crucial role of DCs for the development of clinical autoimmune diseases (Ludewig et al. 2001). The involvement of DCs in autoimmune diseases includes Hashimoto thyroiditis and Grave’s disease, Psoriasis, Sjögren’s syndrome, rheumatoid arthritis and multiple sclerosis (Cravens and Lipsky 2002).

## DCs and CNS autoimmunity

The presence of DCs in the healthy CNS is restricted to the vascular-rich compartments such as the choroid plexus and meninges (McMenamin 1999). DCs can also be detected in the CSF of humans (Pashenkov et al. 2001). Upon local inflammation of the CNS due to infection, cell death or autoimmunity, they are found in the CNS parenchyma (McMahon et al. 2006). There is so far no consensus on whether DCs in the CNS parenchyma come from the periphery (Lande et al. 2008; Zozulya et al. 2010) or may arise from resident microglia (Fischer and Reichmann 2001) and monocytes (Randolph et al. 1998) or whether they migrate from immature DC in the choroid plexus and meninges. The problem arises from the common surface markers on macrophages, microglia and DC subpopulations as well as that they all require the same survival factors in cultures (McMahon et al. 2006). Whatever the origin of DCs in brain parenchyma may be, it has been shown that DCs recruited to the inflammation sites in CNS maintain their ability to migrate to the periphery with CNS autoantigens and prime naïve T cells (de Vos et al. 2002; Karman et al. 2004; Kivisakk et al. 2004).

Involvement of DCs has been described in rodents with EAE, an animal model that resembles MS in humans, where they are discussed as the likely candidate for the initiation and progression of autoimmune reactions by T cells (McMahon et al. 2006). Studies showed that an expansion of DCs following Flt3-ligand treatment (Flt3L/CD135, a growth factor that regulates proliferation of early hematopoietic cells) is associated with enhancement of clinical symptoms and increase of T cell and DCs infiltrates in CNS (Greter et al. 2005). On the other hand, a reduction of DCs after Flt3-L inhibition has been shown to correlate with reduction of severity of disease (Whartenby et al. 2005).

Elevated numbers of DCs that secreted pro-inflammatory cytokines were found in peripheral blood of humans suffering from MS (Huang et al. 1999). Also in CSF, increased numbers of DCs were observed and correlated with common factors of CNS inflammation (Pashenkov et al. 2001). Although active recruitment and accumulation of DCs into CNS lesions of MS patients (Lande et al. 2008) as well as alterations in the interaction between DCs and T cells in MS patients have been reported (Stasiolek et al. 2006), details in the involvement of DCs in MS are so far unknown.

## Autoimmunity in Parkinson’s disease

The destruction of dopaminergic neurons in PD has been connected to a variety of factors, including genetic, environmental and immunologic conditions. Genetic factors have been identified in familiar forms of PD, which contribute to about 10 % of PD cases (Lesage and Brice 2009; Rosner et al. 2008), and pesticides have been identified as environmental risk factors in PD pathogenesis (Liu et al. 2003; Uitti and Calne 1993). Moreover, intravenous drug abuse with meperidine-related substances contaminated with 1-methyl-4-phenyl-1,2,5,6-tetrahydropyridine (MPTP) triggers acute destruction of dopaminergic neurons and PD (Langston et al. 1983). In the past decade, evidence for an immunologic background of PD has been accumulated, on which we will focus here.

Several studies show that PD pathogenesis is associated with neuroinflammation (McGeer and McGeer 2004), which is the prerequisite for the maturation of DCs and their migration to the respective sites in the brain. Following these steps, DCs could be able to trigger an autoimmune response by transferring brain antigens into the cervical lymph nodes and presenting them to T- and B-cells. A potential link between Parkinsonism and autoimmunity has been reviewed by Benkler et al. (2009). Early experimental evidence in favor of an autoimmune background of PD came from Chen et al. (1998) who reported that the transfer of plasma antibodies isolated from PD patients to the substantia nigra of rats induced a marked loss of dopaminergic neurons. In contrast, animals treated with antibodies from healthy controls exhibited much lower neuronal damage, suggesting that autoantibodies that recognize dopaminergic cells are present in patients with PD (Chen et al. 1998). In the last decade, several autoantibodies directed at antigens associated or related to PD pathogenesis have been identified in PD patients, including antibodies directed at melanin (Double et al. 2009), α-synuclein (Papachroni et al. 2007; Yanamandra et al. 2011), and GM1 ganglioside (Zappia et al. 2002). Reversible Parkinsonian syndrome together with the presence of anti-neuronal antibodies has been observed in an EBV-infected patient (Roselli et al. 2006). Autoreactive antibodies associated with PD have not only been found in plasma but also in brain: post-mortem analysis of brains from PD patients and controls showed binding of IgG to dopaminergic neurons in tissues from patients with PD (Orr et al. 2005).

**Fig. 1:**
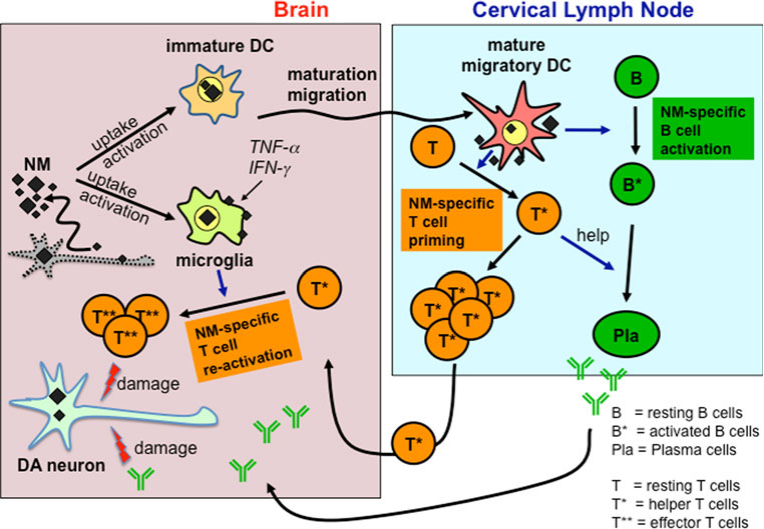
How activation of DCs by NM could trigger autoimmunity directed at dopaminergic neurons. Contact of DCs with NM triggers maturation of these cells that subsequently migrate from the brain into the cervical lymph nodes where they present NM to B- and T-lymphocytes. If NM-reactive lymphocytes are present, they get activated (primed) and secrete NM-specific antibodies (B cells) or exert NM-specific cytotoxic functions (T cells). Activation of microglia by NM would result in a proliferation of NM-specific T cells after contact with NM-presenting microglia. NM-specific antibodies and T cells may recognize NM-positive neurons and trigger their degradation

One potential target structure for an immune attack against dopaminergic neurons is the pigment neuromelanin (NM) that accumulates in dopaminergic neurons as a byproduct of catecholamine metabolism from oxidative polymerization of dopamine and norepinephrine to quinones (Graham 1979). We described recently that NM triggers maturation of DCs in vitro and that this maturation is functional as NM-treated DCs were able on their turn to trigger a proliferative T response. We also showed that DCs can phagocytoze NM (Oberlander et al. 2011). These experiments demonstrate that the first necessary criteria for DCs to initiate an adaptive autoimmune response directed against NM-associated structures are fullfilled. As depicted in Fig. 1, we hypothesize that activated DCs migrate from the brain into the cervical lymph node where they present the potential (auto-) antigens to T and B cells. The recognition of NM as a pathogen or dangerous molecule and its uptake by DCs would allow DC migration and its presentation in the cervical lymph nodes, thereby triggering an adaptive autoimmune response if NM-reactive T or B cells are present. This autoimmune response against NM would be directed against NM-rich cells in the brain, leading to dopaminergic cell death (Fig. 1). This auto-aggressive loop would be enhanced by a NM-triggered activation of microglia, which has been described before (Wilms et al. 2003; Zhang et al. 2011), resulting in an amplification of the adaptive immune response against NM and the local reactivation of immigrating effector T cells (Fig. 1). There is accumulating evidence for an immunogenic role of NM in PD pathogenesis: In sera from PD patients antibodies directed at catecholamine-based melanins have been detected (Double et al. 2009). Moreover, post-mortem analysis of brains from PD patients reveals the opsonization of NM with complement C1q (Depboylu et al. 2011), indicating that NM is recognized by the classical complement pathway as a target structure and shows the capacity to cause neuroinflammation (McGeer and McGeer 2004). Opsonization with C1q is either mediated by previous antibody coating of the target structure followed by recruitment of C1q to the Fc-part of the antibody or by direct binding to C1q ligands (Kojouharova et al. 2010). It remains to be elucidated whether C1q-binding of NM is antibody-dependent or independent and to what extend this complement binding contributes to neuronal cell death. The relevance of the complement system in providing “danger transmitters” to evoke immune responses following danger signals has been discussed thoroughly elsewhere (Kohl 2006). In addition to an immune response directed at NM itself, the high protein affinity of NM (Zecca et al. 2000), together with the efficient phagocytosis of NM by DCs (Oberlander et al. 2011) would allow a DC-mediated presentation of neuronal proteins to the adaptive immune system that are primarily unrelated to NM. In this scenario, NM would act like a Trojan horse, providing access of otherwise unrecognized brain proteins to the DC-triggered adaptive immune response.

## Conclusions

The past decade has provided accumulating evidence for a significant role of the immune system in PD pathogenesis, be it either through inflammation or by an autoimmune response. Thus, immunomodulating therapy strategies aiming to attenuate PD disease progression become an attractive option and warrant further investigation.
